# Practical, Microfabrication-Free Device for Single-Cell Isolation

**DOI:** 10.1371/journal.pone.0006710

**Published:** 2009-08-21

**Authors:** Liang-I Lin, Shih-hui Chao, Deirdre R. Meldrum

**Affiliations:** Center for Ecogenomics, The Biodesign Institute, Arizona State University, Tempe, Arizona, United States of America; The Research Institute for Children, United States of America

## Abstract

Microfabricated devices have great potential in cell-level studies, but are not easily accessible for the broad biology community. This paper introduces the Microscale Oil-Covered Cell Array (MOCCA) as a low-cost device for high throughput single-cell analysis that can be easily produced by researchers without microengineering knowledge. Instead of using microfabricated structures to capture cells, MOCCA isolates cells in discrete aqueous droplets that are separated by oil on patterned hydrophilic areas across a relatively more hydrophobic substrate. The number of randomly seeded *Escherichia coli* bacteria in each discrete droplet approaches single-cell levels. The cell distribution on MOCCA is well-fit with Poisson distribution. In this pioneer study, we created an array of 900-picoliter droplets. The total time needed to seed cells in ∼3000 droplets was less than 10 minutes. Compared to traditional microfabrication techniques, MOCCA dramatically lowers the cost of microscale cell arrays, yet enhances the fabrication and operational efficiency for single-cell analysis.

## Introduction

Single-cell analysis is important for the understanding of cellular genomic regulation [1]. Large arrays of microchambers to culture cells have been the key components of high-content, single-cell level bioassays. The published applications of these assays include the monitoring of cellular gene expression [2], drug screening at single-cell levels [3], viability studies under micro-environmental control [4], [5], monitoring of intercellular interactions [6], and measurement of single-cell respiration rates [7], [8]. Because of the needs of small cell-housing chambers in arrayed formats, the manufacture of these assays to date requires intense microfabrication processes. Arrays of single-cell level microchambers have been fabricated on optical fiber bundles [2], glass slides [5], [7], [8], polydimethylsiloxane (PDMS) [3], and hydrogel [6]. In these studies, cells were seeded by flooding cell suspensions on the arrays and letting cells fall randomly into the microwells. The expected cell occupancy is characterized by Poisson distribution, irrespective of the microwell geometry or surface cell-adhesion treatments unless cell seeding events affect the seeding of other cells [9]. These existing designs have only been applied to the analyses of large eukaryotic cells. Applying these approaches for prokaryotic cells is challenging due to their small sizes; no successful prokaryote seeding of microwells has been reported in the literature to date. An alternative approach is to seed cells in dispersed aqueous droplets of oil-based emulsions [10]–[12]. The generation of droplets depends on deliberate cell and liquid manipulations using microfluidic channel circuits. The volume and density of droplets are controlled by the flow rates of cell medium and oil, regardless of the properties of the seeded cells, so this approach has been applied to both eukaryotic and prokaryotic cells. The statistics of cell occupancy using this approach are also characterized by Poisson distributions [13]. Although powerful, all the above designs are not readily accessible for biologists due to the constraints of microfabrication.

In this paper, we present a simple, yet efficient method for biologists to perform high-content analyses on single or small numbers of cells. This approach does not require any additional engineering or fabrication experience or training. The method, the Microscale Oil-Covered Cell Array (MOCCA), is a large array of compartments for single-cell analysis. MOCCA is a structural, surface-adhering droplet array that preserves the advantages of both solid microwell arrays and droplet-based cell assays. The locations and sizes of the droplets are controlled by hydrophilic patterns on a more hydrophobic surface. Such an array can be made on a common microscope glass cover slip. This fact allows for the observation of cellular details using optical microscopy. In addition, there is no need for microfabrication. We demonstrated the use of MOCCA by isolating *Escherichia coli* (*E. coli*) in picoliter-scale droplets with a single-cell level of occupancy. Our results show that single-cell seeding and isolation with MOCCA is fast and easy, the required equipment is inexpensive, and the only consumables are common microscope cover slips and a small amount of mineral oil.

## Methods

### Surface treatment for cover slips

**Figure 1:**
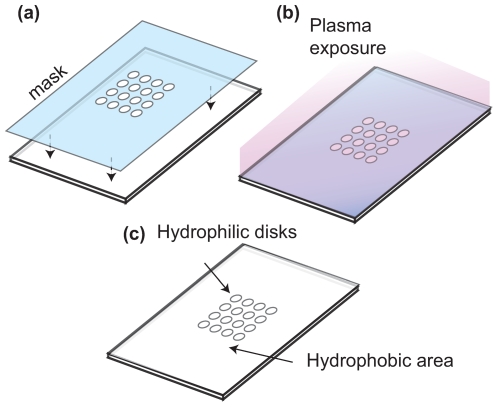
Hydrophilic patterning process. (a) Adhere a mask to a glass cover slip; (b) Expose to air plasma; (c) Remove the mask, leaving a hydrophilic disk array on the surface.

The essential step in the process of creating a MOCCA is to create hydrophilic patterns that define droplet arrays. In this study, such patterns were produced by using microscale PLasma Activated Templating (μPLAT)[14]–[16]. This is a technique that employs a mask to block air plasma exposure in designed areas to increase the hydrophilicity of surfaces. A common 25 mm×25 mm microscope glass cover slip (VWR, West Chester, PA) was used as the substrate for MOCCA. Plasma treatment converted the contact angle of the cover slip surface from 75° to 7°, measured by a goniometer (FTA 1000, Portsmouth, VA). A 142-µm-thick aluminum screen having around 4000 etched holes of 250 µm diameter (Fotofab, Chicago, IL) was used as the μPLAT mask. The mask was set on a cover slip and held in place by its own weight during treatment (Figure 1a). The assembly was then put in a plasma cleaner (PDC-32G, Harrick Plasma, Ithaca, NY) with 6.8 W RF-power for 10 seconds (Figure 1b). The areas exposed to the plasma became more hydrophilic. Finally, the mask was removed, leaving an array of very hydrophilic circular areas on the glass substrate (Figure 1c). The pattern is not visible until droplets are generated and dispersed across the slide.

### Cell and cell medium preparation

*E. coli* were grown in standard Luria-Bertani broth (L.B.) media at 37°C for one hour. The L.B. medium was composed of yeast, tryptone, and sodium chloride with the ratio of 5 g, 10 g, and 10 g, respectively, per liter of medium. The concentration of *E. coli* cells was derived from the optical density (OD) measured by a spectrophotometer (Nanodrop ND-1000, Thermo Scientific, Wilmington), where 1 OD represented 10^9^ cells/mL.

To count the number of bacteria in individual droplets using fluorescence microscopy, the cells were stained with DAPI (4′,6-diamidino-2-phenylindole), a fluorescent material that specifically binds to DNA. 1 mL of broth containing *E. coli* cells were washed with phosphate buffered saline (PBS) and spun down by a centrifuge. Cells were then fixed by mixing with alcohol and allowed to sit for 20 minutes at room temperature. This was followed by another wash with PBS. Then, 0.2 µL of DAPI with a 5 mg/mL concentration was added to 1 mL of cells and the mixture was left at room temperature for 30 minutes. Finally, cells were spun down and then resuspended in PBS. To avoid photobleaching, DAPI-stained cells were kept in a dark refrigerator at 4°C.

### MOCCA formation and cell loading

**Figure 2:**
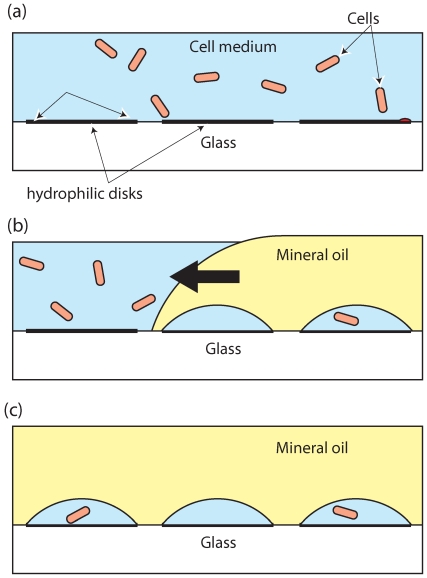
MOCCA formation procedure. (a) Flood the μPLAT-patterned cover glass with cell medium; (b) Flood the cover glass using mineral oil to repel cell medium. Small volumes of medium still occupy hydrophilic disks on the treated surface; (c) After completion, some droplets in the array contain cells.

The procedure for droplet array formation and cell loading is illustrated in Figure 2. A 25 mm×25 mm×2 mm PDMS frame with a 15 mm×15 mm opening is pre-adhered around the hydrophilic disk array to prevent liquids from spilling out of the cover slip. 2 µL of cell medium was pipetted to flood the top of the μPLAT-treated glass (Figure 2a), followed by pipetting ∼5 µL of mineral oil (Sigma M5904) at one side of the glass cover slip. The oil was manually manipulated in such a way as to push the cell medium on top of the array of hydrophilic disks (toward the left in Figure 2b) at a speed of ∼5 mm/second while keeping the cover slip horizontal. The cell medium that originally occupied the untreated background was then replaced by mineral oil, while the medium on plasma-exposed disks formed droplets beneath the oil (Figure 2c). The numbers and locations of droplets correspond to the design of the μPLAT mask. The result was an array of droplets on a glass substrate covered with mineral oil.

## Results

### Array formation

**Figure 3:**
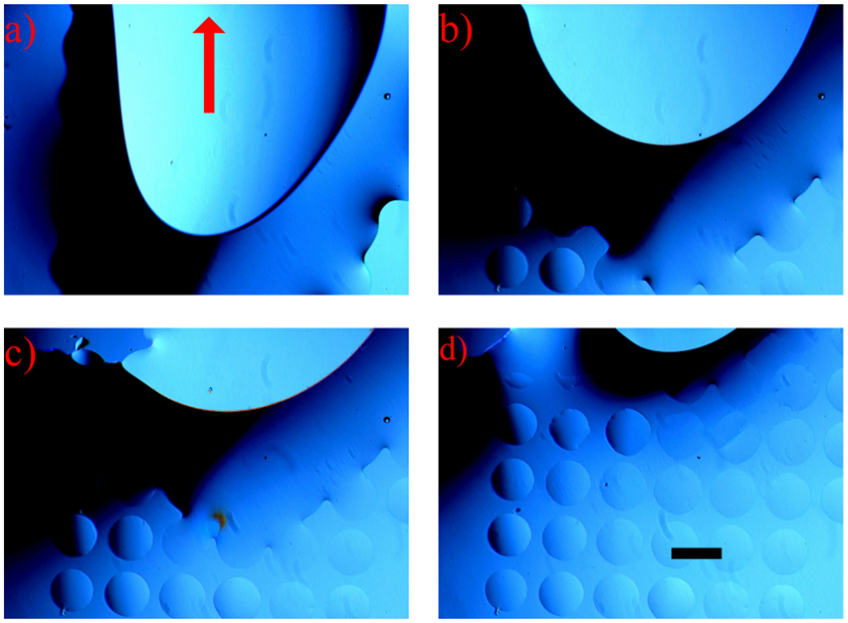
MOCCA generation visualized using an optical microscope. (a) Mineral oil is dispensed around the cell medium (top-center of figure), forcing cell medium to move up; (b–c) Oil does not repel the medium that occupies the hydrophilic areas; (d) When the bulk cell medium has fully retreated, an array of medium droplets is separated by oil. Scale bar in (d) equals 400 um and applies to all pictures.

Figure 3 shows the sequence of MOCCA generation. After loading cell medium on the μPLAT-treated glass cover slip, mineral oil is loaded and forces the cell medium towards the top of the image in Figure 3a. Oil at the bottom of Figure 3a is dragged towards the top using a pipette tip. The medium gradually forms droplets beneath the oil in the hydrophilic areas (Figure 3b). When the bulk cell medium is fully removed, an array of *E. coli-*containing droplets is generated (Figure 3c–d). The mineral oil not only isolates droplets, but also protects droplets from contamination and evaporation. The droplet array in Figure 3d shows small diameter variations, which are associated with the quality of the surface cleanliness and the μPLAT mask roughness. We did not find variations relating to medium or oil volumes, loading speed, or the geometry of the whole array within our experimental conditions. The entire process of array formation takes less than two minutes, making this technique efficient as a high-throughput approach to producing a large cell array on a glass cover slip. The number of droplets in an array depends on the μPLAT mask, which in turn controls the size of the hydrophilic areas. In this example, the MOCCA contained 3000∼4000 droplets on one 25 mm×25 mm glass cover slip, created using a mask of 250-µm-diameter holes.

### Droplet dimension

Assuming the topography of a droplet is a perfect spherical cap, the droplet volume is determined by its diameter (defined by the pattern of the μPLAT mask) and height. The height of a typical droplet was 28 µm measured using a modified microscope to view it from the side. The diameter was 280 µm calculated by viewing the droplet from the top. The droplet diameter was larger than the diameter of mask holes because the surface of the metal mask does not perfectly adhere on the cover glass, leading to a gap between the mask and cover glass. Plasma enters this gap, making hydrophilic disks larger than the holes on the mask. The estimated droplet volume was calculated as 887 picoliters. Compared with the sizes of typical eukaryotic cells and prokaryotic cells, the volume of the droplet was deemed suitable for single cell analysis.

The variation of the droplet volumes is an important subject, but using a side-viewing microscope to profile a large number of small immersed droplets is labor-intensive and impractical. Instead, we measured the variation indirectly by loading a droplet array of homogenous fluorescein solution (1 mg Fluka 46955 powder in 100 mL of water) and imaged the total fluorescent intensity of each droplet. Assuming the fluorescent intensity is proportional to the droplet volume, the variation of total fluorescent intensity implies the variation of volume. The resulted standard deviation of fluorescent intensity is 23% relative to the mean intensity, measured from 144 droplets on one MOCCA array. If a typical droplet has 887 picoliters, the droplet volume is determined to be 887±201 picoliters.

### Effects to image quality

**Figure 4:**
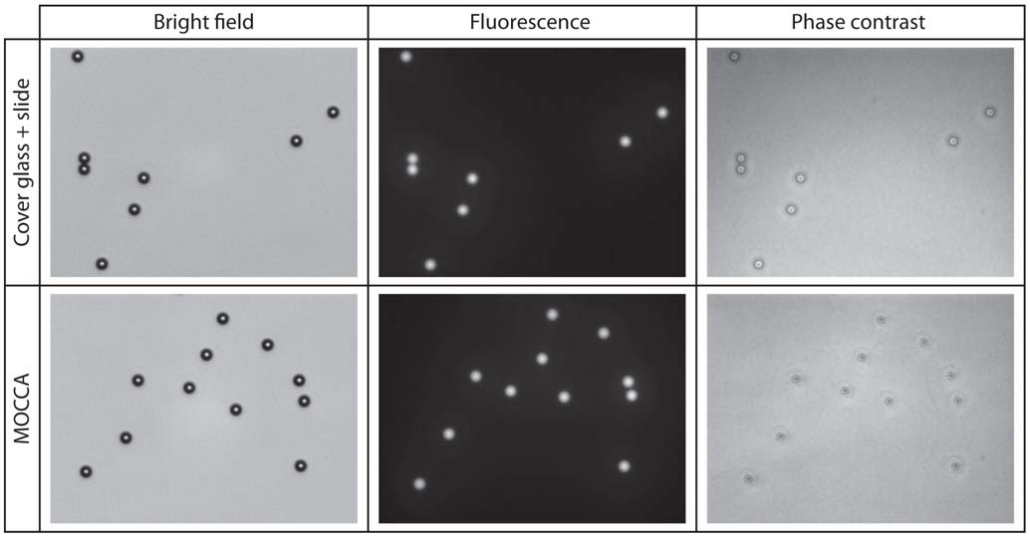
Result comparison of microscopy techniques between using conventional microscope slide with cover glass and MOCCA.

The droplets on MOCCA do not affect bright field and fluorescence microscopy, but the spherical water-oil interface interferes phase contrast imaging. To illustrate the optical effects of MOCCA, we used the conventional specimen configuration as the “gold standard”, where the specimen was sandwiched by a microscope slide and a cover glass, with the cover glass facing toward the objective (Nikon Plan Fluor ELWD NA0.85 Ph2 60×). 5-µm green fluorescent microspheres (G0050, Thermo/Duke Scientific, Fremont, CA), a standard tool for imaging system characterization, were seeded into the droplets following the procedure in Fig. 2 as the calibrant to verify image quality. Figure 4 compares the results of various images taken on conventional slides and MOCCA, with identical camera and optical settings for each imaging methods. No difference can be observed from the bright field images and fluorescence images, indicating imaging using these techniques on MOCCA is very similar to using conventional slides. However, the quality of the phase contrast image is worse for the MOCCA case.

### Cell loading

**Figure 5:**
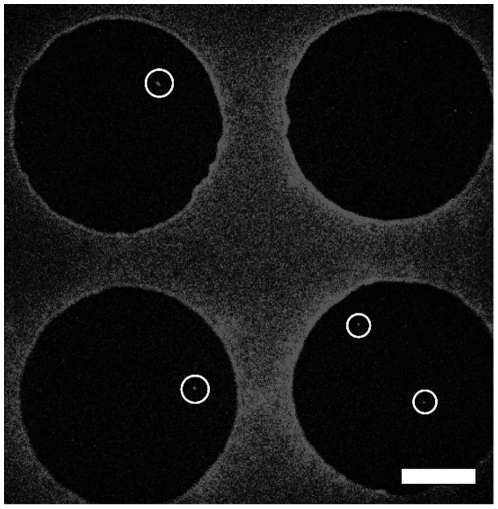
DAPI-stained *E. coli* on MOCCA. Two droplets in the field of view contain one cell (highlighted with circles). Scale bar equals 100 µm.

DAPI-stained *E. coli* cells were loaded in the cell array based on the procedures described previously in the experiment section, and the number of *E. coli* cells in each droplet was manually counted on a fluorescent microscope (Nikon TE2000-E). Figure 5 shows DAPI-stained *E. coli* cells in four droplets on MOCCA. A 20× objective (Nikon Plan Fluor ELWD NA0.45 20×) was used to view the entire droplet, while allowing single DAPI-stained cells to be identified at the same time. The small bright spots inside the highlighted circles are *E. coli* cells. Because DAPI adheres differentially to more hydrophobic surfaces, we were able to use the DAPI-adhered fluorescent background to outline the footprint of the droplets in Figure 5. With a starting concentration of 2.1×10^6^ cells/mL, two droplets contained single cells, one contained two cells, and the other was empty.

### Control of cell distribution

**Figure 6:**
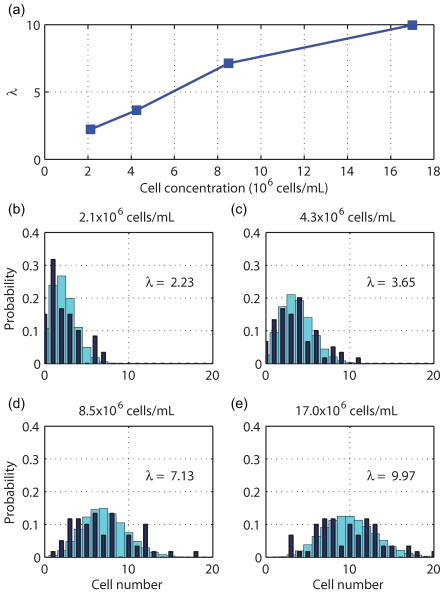
Control of cell distribution on MOCCA. (a) The average cell number (λ) per droplet versus experimental bulk cell concentrations. (b–e) Cell distributions of four cell concentrations in bulk medium. Dark bars: experimental results; light bars: fit Poisson distribution.

The overall cell occupancy in droplets is a function of the initial bulk cell concentration. High initial concentrations result in multiple cells in a high percentage of droplets, whereas low initial concentrations result in empty droplets. The initial cell concentration for single-cell seeding is in the order of one cell per droplet volume. For the 900-pL droplets in this study, a rough estimate of the cell concentration needed for single-cell occupancy is around 10^6^ cells/mL (∼1 cell/900 pL). To empirically characterize the relation between initial bulk cell concentration and cell occupancy, four different concentrations of DAPI-stained *E. coli* cells (17.0×10^6^, 8.5×10^6^, 4.3×10^6^, and 2.1×10^6^ cells/mL) were used to seed MOCCA. In these experiments, cell occupancy was measured by counting the cell number in 60 randomly chosen droplets using a fluorescent microscope for each tested cell concentration. The observed average cell occupancy (denoted *λ*) is the mean of the counted cell numbers in each selected droplets. Figure 6a shows that *λ* is roughly proportional to the bulk initial cell concentration.

For one initial cell concentration, cell occupancy is a discrete Poisson random variable. The probability *f* for *n* cells to be seeded in a droplet is
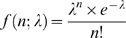
(1)


The experimental cell occupancy distributions of the four tested concentrations are shown in Figures 6b–e as histograms in dark bars. The light bars in these figures are the Poisson distribution from Eq. (1) , where *λ* is substituted with the average cell occupancy of each cell concentration. The experimental and theoretical distributions match reasonably well. The experimental histogram for the 2.1×10^6^ cells/mL concentration case has a distinct, 32% high peak at 1 cell/droplet. The average cell occupancy *λ* is 2.23 cells/droplet, approaching single-cell levels.

## Discussion

We present a unique approach to a cell array using a simple method to generate a microscale oil-covered cell array (MOCCA) on a glass cover slip. We demonstrate this approach using commercially available glass cover slips, mineral oil, metal screens, and a plasma cleaner. Furthermore, the process is shown to encapsulate *E. coli* in a large array of droplets close to single-cell levels. Cell loading has been shown to be a Poisson process, where the cell numbers in droplets can be well-controlled through cell concentration. By utilizing conventional cover slips, the MOCCA is especially useful for single cell studies using inverted fluorescence. Compared to traditional microfabrication techniques, MOCCA dramatically reduces the cost and turn-around time for fabrication of single cell arrays, making it affordable for researchers. Although the planar dimensions of droplets are limited by the dimensions of the μPLAT mask (250 µm in our study), the droplet volumes (∼900 pL) are still suited for single cell studies. Although the presented method aims to provide an assessable platform for researchers, MOCCA can be integrated into microfluidic chambers with inputs for cell medium and oil to facilitate automated, high-throughput analyses.
